# Efficiency of Polyethylene Terephthalate Glycol Thermoplastic Material to Functional and Expansion Forces in Orthodontic Applications: An Experimental Study

**DOI:** 10.1155/tswj/7232779

**Published:** 2025-01-10

**Authors:** Anosh A. Haik, Yassir A. Yassir

**Affiliations:** ^1^Department of Orthodontics, College of Dentistry, University of Baghdad, Baghdad, Iraq; ^2^Department of Orthodontics, School of Dentistry, University of Dundee, Dundee, UK

**Keywords:** 3-point test, expansion, functional forces, mechanical test, twin block

## Abstract

**Background:** While polyethylene terephthalate glycol (PETG) is widely used in orthodontic appliances such as clear aligners and retainers, there is limited experimental data assessing its performance under functional stresses, such as those encountered during dental movements and palatal expansion.

**Objective:** This study aims to evaluate the ability of PETG thermoplastic material to withstand deformation under functional and expansion forces, specifically within the context of orthodontic applications.

**Subjects and Methods:** To estimate the firmness of the screw within the appliance, a universal Instron testing machine was used to record the forces released by each activation of the expander within the upper part of 10 clear modified twin blocks (MTBs) made from PETG and compare it with that released by 10 conventional twin blocks (CTBs). On the other hand, to determine the ability of the thermoplastic appliance to withstand the deformation during functional forces, a three-point bending test was used to investigate the response of both appliances under static loading. Independent samples *t*-test was used to compare the differences between groups.

**Results:** Both CTB and MTB groups follow the same pattern of increase and decrease in the amount of mean load with the CTB group line showing a considerably higher amount of mean load reaching the peak (334.5 N) at turn 25 of screw activation while the peak of mean load for MTB group was equal to 252.6 N at turn 23. There was a statistically significant difference between the CTB and MTB groups in the three-point bending test (*p*=0.001). However, both appliances did not deform at the required force.

**Conclusions:** The MTB can withstand both required expansion and functional load without deformation.

**Trial Registration:** ClinicalTrials.gov identifier: NCT06116500.

## 1. Introduction

Orthodontic appliance design has evolved significantly to accommodate patient preferences for better aesthetics during treatment. This progression led to the development of invisible or esthetic orthodontic options, such as ceramic brackets, lingual appliances, and removable clear thermoplastic appliances. Clear appliances have gained popularity due to their invisibility, simplicity, hygiene, comfort, and reduced impact on mastication [1–3].

Removable clear appliances are typically manufactured either through thermoforming (a process that shapes thermoplastic sheets into three-dimensional (3D) forms using heat, vacuum, and pressure) or through 3D printing, which uses photo-polymerization of clear liquid resin [4, 5]. The thermoforming process remains the most widely used for producing clear appliances, either from pure thermoplastic sheets (composed of a single material) or blended sheets (composed of two or more materials) [6, 7].

Thermoplastics, synthetic polymers that melt when heated and harden upon cooling, offer a range of mechanical properties that depend on their molecular design and environmental conditions, such as temperature and humidity. These materials can be classified as noncrystalline (amorphous) such as polycarbonate (PC), polyurethane (PUR), and polyethylene terephthalate glycol (PETG), or crystalline, such as polypropylene (PP) and polyethylene (PE) [8]. In dental applications, thermoplastics undergo rigorous safety testing and are evaluated for compliance with international standards, including American Society for Testing and Materials (ASTM) or International Organization for Standardization (ISO) certifications. Their use as an alternative to acrylic in dental devices continues to expand [6, 9].

PETG is an amorphous polymer produced by the polycondensation of ethylene glycol with terephthalic acid. The addition of glycol enhances the processability of PET, lowers its crystallization temperature, and improves the material's mechanical properties, resulting in a versatile, colorless, and transparent substance that is widely used in packaging, medical containers, and electronics [10–12]. PETG's desirable characteristics, such as high mechanical strength, good formability, fatigue and abrasion resistance, and dimensional stability in moist environments, make it particularly suitable for orthodontic applications. It is already used in manufacturing retainers [7], splints [13], and tooth aligners, though it has not yet been explored for functional or orthopedic appliances [14–17].

Functional orthodontic therapy aims to correct skeletal malocclusion by encouraging or redirecting the growth of skeletal structures in growing individuals. The twin block, a commonly used removable functional appliance, is versatile and easily modified to suit various treatment needs [18, 19]. One such modification, the clear twin block, integrates self-cured acrylic ramps with clear thermoplastic appliances to enhance aesthetics and improve patient compliance. Earlier studies have used materials like biocryl (pure polymethyl methacrylate [PMMA]) or PC in thicknesses of 1–1.5 mm to fabricate these clear twin block appliances [20–23].

However, no studies have yet investigated the use of PETG thermoplastic for manufacturing twin block appliances, nor has its use been explored for fabricating clear expanders. Given the unique properties of PETG, particularly its 2 mm thickness, this study aims to evaluate the mechanical properties of a modified twin block (MTB) appliance made from PETG compared to a conventional twin block (CTB) made from acrylic. Specifically, the study will assess the appliances' ability to withstand deformation under applied forces and during expansion, providing new insights into the effectiveness of clear PETG appliances.

## 2. Materials and Methods

### 2.1. Study Design

This is an experimental study that was designed as a part of a randomized clinical trial, using two mechanical tests to analyze the compression strain that developed in the modified twin block (MTB) appliance with expander, in comparison with the CTB appliance with expander. Ethical approval was obtained from the ethics committee at the College of Dentistry, University of Baghdad (Reference No. 664 in 13.9.2022).

## 3. Methodology

The main materials used in the fabrication of the appliances and samples for the study are summarized in Table 1. Two mechanical tests were conducted: a mechanical loading test using the three-point bending method and a compression test.

**Figure 1:**
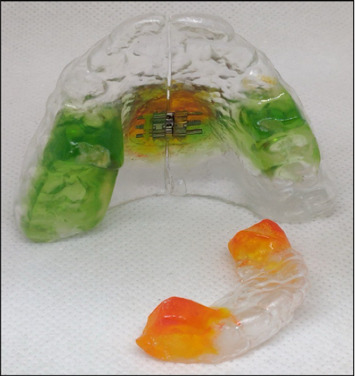
Design of modified twin block appliance with expander.

The MTB appliance with an expander was designed using 2 mm thick biocompatible PETG thermoplastic material. This material was adapted to maxillary and mandibular casts individually using a pressure molding vacuum machine. After molding, the two clear appliances were trimmed, finished, and transferred onto an articulator with the help of a pre-existing working wax bite. Cold-cure acrylic was then used to fabricate the ramps on the thermoplastic sheets. Finally, the expansion screw was positioned in the midline of the maxillary cast, and the maxillary appliance was split midpalatally, as shown in Figure 1.

### 3.1. Mechanical Loading by Three-Point Bending Test

**Figure 2:**
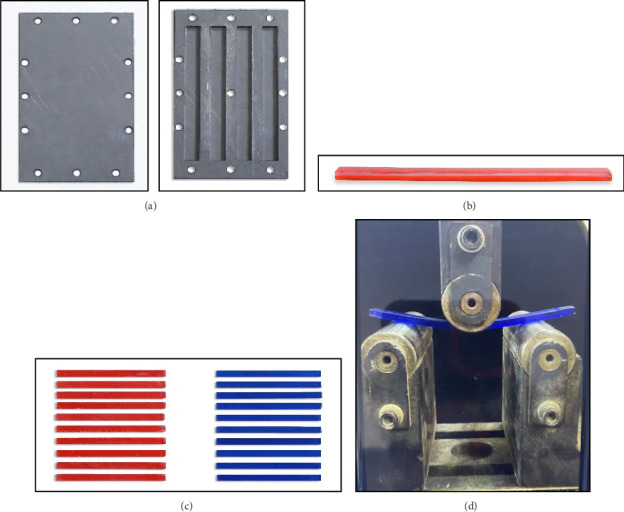
(a) Stainless-steel mold produced by water-jet cutting into four digits according to the ASTM standard (D790-17) of specimen dimension. (b) Cross section of the clear twin block appliance specimen showing upper layer is 2 mm thickness of PETG thermoplastic plate and 1.3 mm is cold-cure acrylic. (c) The 20 specimens, the red ones are clear group, while the blue ones are conventional group. (d) The specimen was placed on the stand and subjected to the load cell.

To determine the ability of the thermoplastic appliance to withstand the deformation during applied forces, a three-point bending test was used to investigate the response of MTB appliances under static loading compared with the CTB appliances. The experiment evaluated 10 samples for each group, including the following steps:

*Preparation of samples*: In 2017, the standard ASTM (D790-17) [24] outlined the preferred dimensions for various types of plastic materials. For plastic specimens, the optimal measurements are 12.7 mm in width, 3.2 mm in thickness, and 127 mm in length. To meet these specifications, a stainless-steel mold was created by using waterjet cutting to carve a 100 × 160 mm stainless-steel block into four individual pieces, allowing for the simultaneous production of four specimens. Each piece was designed to match the precise dimensions of the specimens (Figure 2(a)). Ten specimens from the MTB appliance group were fabricated through the following steps: A 2 mm thick PETG thermoplastic sheet was heated in a thermoforming machine to 220°C until it became soft and pliable. Once the sheet reached the required temperature, it was swiftly placed over a stainless-steel block, and pressure was applied to mold the thermoplastic tightly to the block shape. After cooling and hardening, the molded sheet was removed from the block. The standard dimensions of the specimen were marked on the thermoformed sheet using a marker and ruler (or by tracing one of the prepared acrylic specimens). A carbide fissure bur was used to cut the thermoformed sheet, and the trimmed material was adapted into a stainless-steel mold. Cold-cure acrylic was then applied over the thermoformed sheet using the sprinkle method. Once the material had set, the specimen was carefully removed from the mold (Figure 2(b)). The other 10 specimens belonged to the CTB appliance group and were fabricated entirely from cold-cure acrylic using the sprinkle method. The process began by applying a separating medium over all parts of the stainless-steel mold to facilitate easy removal of the specimens. Cold-cure acrylic was then distilled directly into the mold using the sprinkle method. Once the acrylic had set, the specimens were carefully removed from the mold. After being prepared, the samples were ready for testing (Figure 2(c)).

*Testing of the specimens*: The specimens were subjected to a three-point bending test using the universal Instron testing machine equipped with a 100 kN load cell according to the standard protocol of ASTM (D790-17). The specimen was extended at a rate of 5 mm/min, and data were collected at a frequency rate of 100 Hz. The sample was positioned on a stainless-steel stand that had a rectangular base and two vertical supports with a 100 mm span (apart from each other) and 30 mm curvature radius (Figure 2(d)). Each specimen was subjected to the load cell and load–deflection curve recorded within the built-in software of the machine; the specimen was deflected at a speed of 100 mm/min.

### 3.2. Compression Test

To estimate the firmness of the screw within the appliance, a computer-controlled electronic universal Instron testing machine (LARYEE, UE343000, China) with high accuracy load cell (±0.5% of full scale) and sampling rate up to 100 kHz (kHz). The devise was equipped at 100 kN with full resolution and used to record the forces released by the expander within the upper part of MTB appliance and compare it with the forces released by that of the CTB appliance. Ten appliances were evaluated for each group, including the following steps:

*Preparation of DIA-stone* models: Ideal DIA-stone models with the same intercanine and intermolar distance were prepared similarly to the preparation of ideal study models [25] by mixing 20 mL of water with 100 g of HIRO diastone (following the manufactural directions) in a rubber bowel with a plaster spatula for 30 s with stropping motions. Then, we place the bowel on the vibrator to allow all bubbles to rise to the surface and break. A small amount of mixture was poured into the distal of the last molar side of a silicon mold under the vibrator allowing for a gradual flow of the mixture within teeth spaces until the arch filled with the mixture. Then, with a plaster spatula, all the mixture was added within the plastic mold to make the base of the models, waiting for 10–12 min (loss of gloss of the mixture) for the complete set of the DIA-stone model, and the procedure was repeated 20 times.

**Figure 3:**
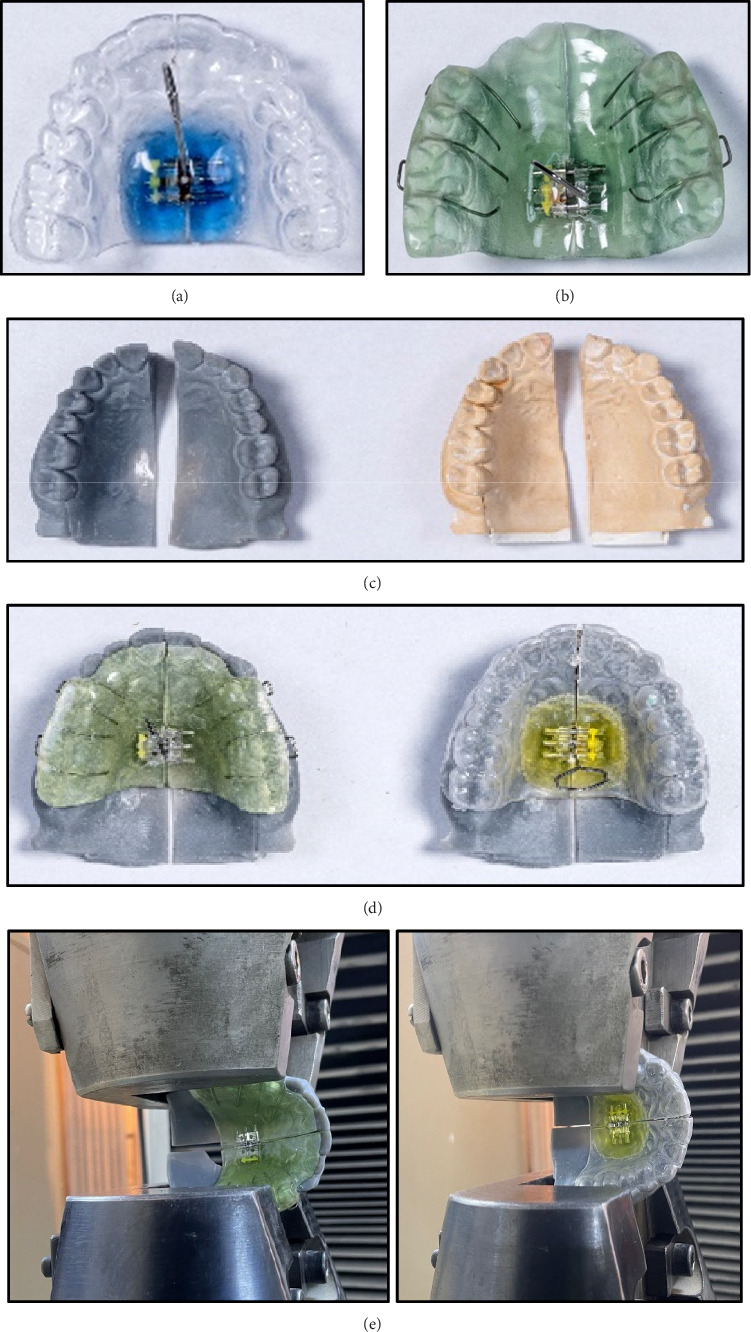
(a) Design of upper part of clear twin block appliance. (b) Design of upper part of conventional twin block appliance. (c) Digital and DIA-stone splitted models. (d) Digital splitted models with testing appliances. (e) The appliance in each group with the splitted digital model grasped in the upper and lower clamps of the Instron machine before starting the activation process.

*Fabrication of Appliances*: Following the basic design of the standard twin block appliance [17, 26, 27], 10 upper parts of twin block appliance were fabricated on the DIA-stone models of each group. For the MTB group appliances, the preparation process in the dental laboratory followed these steps: A 2 mm thick PETG thermoplastic sheet was heated in a thermoforming machine to 220°C until it became soft and pliable. Once heated, the sheet was swiftly placed over a prepared DIA-stone model, and pressure was applied to mold the thermoplastic tightly to the contours of the teeth. After allowing the material to cool and harden, the molded sheet was carefully removed from the model. Excess material was trimmed using scissors, and the edges were smoothed and polished with fine sandpaper and polishing wheels to eliminate sharp edges. A mid-palatal screw was then positioned in the center of the PETG appliance, and cold-cure acrylic was applied using the sprinkle method around the screw's edges to securely integrate it within the appliance. The appliance was split midpalatally and polished (Figure 3(a)). While for the CTB appliance group, the preparation process in the dental laboratory involved the following steps: Adams' clasps were bent on the DIA-stone model to fit the upper permanent first molars and first premolars, serving as retentive components. A mid-palatal screw was centrally positioned on the model at the level of the second premolars. Cold-cure acrylic was then applied using the sprinkle method around the edges of the screw to securely integrate it within the appliance. The acrylic was extended to cover the occlusal surfaces of the upper posterior teeth, ensuring proper fit and stability (Figure 3(b)).

*Preparation of split digital model*: According to the guidelines by Chaconas and Caputo [28] and Oshagh et al. [29], the appliance on a split model was hold by the clamps (specimen grips) of the Instron machine during the testing procedure. A crosscut straight fissure bur was used to create a line in the middle of DIA-stone models, and with grinding disc, the models were split into two halves. These halves were scanned with 3D scanner (Dentsply Sirona inEos X5 scanner, Germany) in order to converting them to a digital file format (STL) and these files sent to 3D printer (Phrozen Sonic Mighty 4K MSLA 3D printer, Taiwan) and printed in a form of 3D split resin digital models (Figures 3(c) and 3(d)).

*Testing of the appliances*: The universal Instron testing machine securely hold the appliance and the split digital mold using upper and lower clamps. The midline screw of each appliance was activated by a key, with each turn producing 0.25 mm of expansion. This compression force was then transmitted hydraulically to the jaw of the Instron machine, and the resulting force was recorded in Newton units by the software of the machine for each turn (Figure 3(e)). This process continued until the screw reached its maximum separation capacity, or there was a decrease in the recorded force. This testing procedure adhered to the standard protocol for estimating the rigidity of the screw in a new appliance design or material [29–31].

### 3.3. Statistical Analysis

Statistical Package for Social Science Version 25.0 (SPSS Inc., Chicago, IL. USA) was used for statistical analysis with statistical significance set at *p* < 0.05.

All responses (Test I and Test II) were collected and saved as an Excel spreadsheet (Excel, Microsoft Office Professional Plus 2019, Washington, USA).

The Shapiro–Wilk test was used to assess the normality of data distribution, while the independent samples *t*-test was used to compare the difference between groups.

## 4. Results

### 4.1. Mechanical Loading by Three-Point Bending Test

Ten samples for each group were tested by a three-point bending test. The descriptive statistics are shown in Table 2. For the samples of the CTB group, the mean value of the maximum load reached 159.5 N (SD 7.62) which is higher than that of the MTB group (145 N, SD 8.5).  Table 3 also shows that the data for both groups were normally distributed (Shapiro–Wilk test). Independent samples *t*-test revealed a statistically significant difference between CTB and MTB groups (*p*=0.001) (Table 2).

### 4.2. Compression Test

Ten appliances for each group were tested by measuring the amount of load (compression forces) produced with each turn of the mid-line screw (each activation of the screw resulted in 0.25 mm of expansion), and the results of the test were described, for 30 turns, as shown in Table 4.

**Figure 4:**
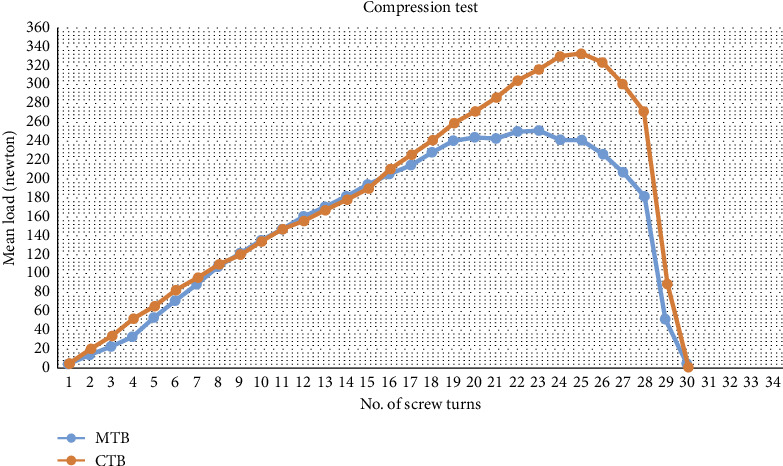
A line chart displaying the load applied on each appliance after each turn of the expansion screw (compression test).

A line chart, as clarified in Figure 4, illustrated the mean value of the applied load (Newton) after each turn of expansion. The initial phase of increase loads up the two lines (of CTB, and MTB groups) to maintain the same pattern with almost the same level of gradual growing in the amount of load till turn 17 of screw activation. Then, the CTB group line showed a considerable increase in the amount of mean load reaching the peak 334.5 N (SD = 43.43) at turn 25 of screw activation while the peak of mean load for MTB group was equal 252.6 (SD = 82) Newton at turn 23. After the peak of the mean value of each group, both lines gradually declined until reaching 0 N loads at turn 30 of screw activation.

## 5. Discussion

Generally, when designing an orthodontic appliance using thermoplastics, it is crucial to select a material that has efficient mechanical properties that can match the appliance needs and treatment requirements within the stimulated intraoral environment [15]. Numerous studies have demonstrated that PETG possesses excellent mechanical properties and has been approved for safe use in the dental field. It is commonly used for fabricating temporary bridges in areas requiring esthetic attention [32] and for creating teeth bleaching trays [33]. PETG thermoplastic foil splints with bonded wire cleats have also been applied in occlusal management. The cleats enable intermaxillary fixation with orthodontic elastics, guiding and maintaining the occlusion in centric relation [34, 35]. Additionally, within simulated oral environments, PETG provides stable forces to target teeth, making it suitable for use in tooth aligners [36–38]. PETG thermoplastics till now not been used in the fabrication of functional appliances, so they were chosen for the construction of the twin block appliance, and for that reason, the current experimental study was conducted to investigate the ability of this thermoplastic material including two mechanical tests.

### 5.1. Mechanical Loading by Three-point Bending Test

The mechanical loading of specific apparatus on certain materials (specifically thermoplastics) is simply and easily tested experimentally by a three-point bending test using a flat material sample of defined size depending on certain standardizations [39, 40]. Hence, a sample with a specific dimension depending to the standard by ASTM (D790-17) [24] in 2017 for thermoplastic (molding) materials was used in this study. For myofunctional appliances, the force magnitude is lower than for orthopedic appliances to enhance patient compliance to wear the appliance and higher than that for removable orthodontic appliances to produce enough force in their action. It had been found that each 1 mm of displacement of the dental base by myofunctional appliance could produce 100 g (1 N) of force that stretches the muscles and may reach 500 g (5 N) depending on the severity of the malocclusion [26, 41].

The CTB group had a mean maximum load value of 159.5 N (SD 7.62), which was higher than the MTB group value of 145 N (SD of 8.5). The CTB appliances were constructed from PMMA. A material characterized by its high modulus of elasticity and rigidity, which contributes to its ability to resist higher load before deformation. The notable stiffness and strength of acrylic were well-documented in orthodontic literature [42], making it a suitable material for appliances that require significant load-bearing capacity. In contrast, MTB appliances are constructed from PETG thermoplastics, a material known with its flexibility and lower modulus of elasticity compared to the acrylic. This characteristic means that PETG deforms more readily under load, allowing it to absorb and dissipate energy rather than resist it rigidly. Accordingly, the MTB samples exhibited a lower mean maximum load in the bending test. These results are consistent with findings by Albertini et al. [43], which demonstrated lower force values for PETG thermoplastics compared to other thermoplastic materials. On the other hand, the PETG ability to evenly distribute stress across the surface of the appliance could likely enhance its long-term durability. As highlighted by Lombardo et al. [38] and Elkholy et al. [44], PETG has a certain degree of stiffness and excellent stress relaxation properties allowing it to gradually return to its original shape after deformation, which reduces the likelihood permanent damage and increases the appliance lifespan in clinical settings. The ability of PETG to undergo more significant deformation before failing makes it less prone to breaking under sudden or high loads, which can be beneficial in clinical scenarios where the appliance may be exposed to variable forces.

Although there were statistically significant differences between the two groups, the amount of load applied on the MTB group was still much greater than the required range of functional force magnitude for myofunctional appliances to withstand the MTB without deflection. In the current test, the balance between strength and flexibility of PETG was in line with other studies of three-point bending test [38, 43–45]. This gives the PETG an advantage of being fabricated and designed appropriately with lower risk of appliance breakage under masticatory forces by reducing the localized pressure points.

### 5.2. Compression Test

The design of twin block appliance in this study included a screw (orthodontic expander) to provide a functional dentoalveolar expansion to correct positional crossbite which could happen after the mandibular protrusion [46–49]. Based on the compression test results and the line chart presented in Figure 4, the initial phase of increase loads reveals that the distribution of expansion force was even across both appliance structures. The difference in peak values reflects that the CTB appliance might provide greater resistance against the applied forces with higher stress maintenance within the acrylic. This in turn may lead to increased force requirement for expansion and exposing the appliance to the risk of fracture. The lower peak value of the MTB may reveal a better force distribution and lower resistance to expansion and consequently reduce the risk of appliance fracture. The gradual decline in the applied force for both appliances explains that as the expansion progress the resistance will reduce.

This indicates that in spite of the resiliency of cold-cure acrylic (for CTB group) being higher than PETG thermoplastics (for MTB group), both groups have the ability to withstand the optimal delivered dentoalveolar expansion force (8.9–17.8 N) multiple times, with an expansion rate of 0.5–1 mm per week. The amount of force delivered per expansion was reported by several authors [50–52]. It was evident from this test that PETG has high mechanical strength which allows it to withstand applied loads for a significant period of time before undergoing plastic deformation. This could be attributed to the superior stress relaxation property of the PETG in comparison with that of the acrylic which is essential for ensuring uniform dentoalveolar movement and minimize stress concentration [11, 14].

In both tests, the PETG thermoplastics demonstrated promising thermal bonding with acrylic, as there was no separation occurred between the two materials under the applied load. Moreover, the PETG material exhibited high flexibility with minimal deflection. The peak mean load values for both tests were 252.6 N and 145 N, respectively, which exceeded the required level of expansion and functional load during treatment. These findings suggest that glycol, which is present in the chemical composition of PETG thermoplastic material, plays an important role in enhancing the material robustness and mechanical properties [10, 12]. Additionally, PETG thermoplastics have a significant stress relaxation characteristic, meaning it slowly converts elastic strain into plastic strain even below the yield strength levels. This property is closely linked to PETG viscoelastic property [31]. During the compression test, the MTB group showed a gradual reduction in the applied mean value load similar to the CTB group, indicating this property. However, it was not as apparent in the three-point bending test due to the insufficient time for plastic deformation of the specimens during measurement.

For optimal stress distribution and to maintain the viscoelastic properties of thermoplastic material, it is recommended to increase its thickness [16]. A thinner material leads to lower yield and tensile strength, making it more prone to deformation and fractures [53]. To ensure the PETG material can withstand functional treatment force without breaking and maintain its elastic and viscous behavior during plastic deformation and to allow stress distribution over a larger area, a thickness of 2 mm was chosen as compared with previous studies that used other types of thermoplastics from a 1–1.5 mm thickness [22, 23].

The findings of this study are concordant with studies on mechanical properties of PETG in orthodontic appliances [54–57] supporting that the use of PETG for its balance of strength and flexibility reinforces its suitability for clinical use in clear aligner and other orthodontic appliances.

This study provides an insight on a novel appliance that could function properly compared with the twin block in addition to greater potential to be acceptable by the patients due to its invisibility, easiness of insertion/removal, and flexibility. However, based merely on two mechanical tests may not be sufficient to understand its intraoral behavior as a functional appliance. Therefore, a randomized clinical trial is required to test its effectiveness.

## 6. Conclusions

Both the CTB and MTB groups display similar patterns of resistance when subjected to expansion load delivered by the orthodontic expander (screw). This load is several times greater than the ideal dentoalveolar expansion force. The MTB can withstand the required functional load without deformation.

### 6.1. Limitations of the Study

A larger sample size would provide more robust statistical analysis and enhance the reliability and generalization of the results. Variations in environmental conditions of oral cavity, such as temperature and humidity, could influence the mechanical properties of the thermoplastic material. Conducting tests under standardized conditions may mitigate this limitation. Moreover, since the study was performed in an experimental setting that provides valuable insights into parts of mechanical properties of PETG material, such findings may vary when applied in the oral cavity due to exposure to masticatory forces and different physical and chemical factors. Short duration evaluation of the study might not capture potential changes or degradation that could occur over extended periods of clinical use. The direct clinical relevance and performance of this material in orthodontic treatments may require further investigation through in vivo studies or clinical trials.

### 6.2. Clinical Implications

The study found that PETG thermoplastic material displayed strong thermal bonding with acrylic, indicating its potential for clinical use. It also showed adequate stress relaxation, and balance between flexibility and rigidity. The peak mean load values exceeded required levels for expansion and functional treatment, indicating its suitability for this orthodontic application. The robustness, flexibility, and significant stress relaxation of PETG also exhibit a property essential for orthodontic appliances. Increasing material thickness is recommended for optimal stress distribution and maintaining viscoelastic properties. These findings suggest that PETG thermoplastic material could be a valuable choice for orthodontic treatments, offering durability and reliability.

## Figures and Tables

**Table 1:** Materials used in the fabrication of the sample.

Materials	Trade name	Manufacturer	Lot. Number
Type 4 modified synthetic dental die stone	HIRO HardRock®	Mutsumi Chemical Industries, Romania	ISO 6873
Hard stainless-steel Ø 0.70 mm wire	remanium® draht/wire	Dentaurum Dental Product, Germany	446,380
Biomedical stainless-steel standard screw for upper removable orthodontic appliances 9 m	Leone® standard expansion screw	Leone Spa Orthodontics and Implantology, Italy	A0805-96
Methyl methacrylate monomer and methyl methacrylate polymer	Orthocryl®-liquid (monomer), and Orthocryl® EQ-powder (polymer)	Dentaurum Dental Product, Germany	Liquid (492,691), powder (519,245)
Polyethylene terephthalate glycol (PETG) transparent thermoplastic plates, 125∗125 mm, thickness 2 mm	Leone® thermoforming material	Leone Spa Orthodontics and Implantology, Italy	ISO 10993-1

**Table 2:** Descriptive statistics for the maximum load applied (Newton) during the three-point test and testing the normality of data distribution (Shapiro–Wilk test).

Group	*N*	Minimum	Maximum	Mean	Std. deviation	Std. error mean	Shapiro–Wilk *p* value
CTB	10	150	170	159.50	7.62	2.41	0.089
MTB	10	135	160	145.00	8.50	2.69	0.200

**Table 3:** Independent samples *t*-test for the difference between groups in a three-point test.

Group	Levene's test for equality of variances		*t*-test for equality of means			Mean difference	Std. error difference	95% confidence interval of the difference	
	*F*	*p*	*t*	df	*p*				
								Lower	Upper
CTB-MTB	0.017	0.899	4.017	18	0.001	14.50	3.61	6.92	22.08

**Table 4:** Descriptive statistics for the maximum load applied (Newton) during the compression test. “T” stands for the number of screw turns.

Group	No. of screw turns	*N*	Minimum	Maximum	Mean	Std. deviation
CTB	T1	10	2	8	4.20	2.20
MTB		10	2	4	2.80	1.03

CTB	T2	10	10	26	19.20	5.75
MTB		10	10	18	13.60	3.24

CTB	T3	10	18	52	34.60	11.96
MTB		10	16	34	23.40	5.34

CTB	T4	10	34	70	52.00	11.20
MTB		10	20	60	34.80	11.32

CTB	T5	10	44	82	66.80	13.41
MTB		10	30	86	52.40	16.70

CTB	T6	10	52	106	82.20	17.19
MTB		10	40	114	72.20	22.24

CTB	T7	10	60	124	95.00	19.51
MTB		10	50	134	87.00	25.65

CTB	T8	10	72	136	108.60	22.37
MTB		10	64	160	107.60	28.03

CTB	T9	10	80	156	119.40	22.49
MTB		10	74	174	121.40	28.86

CTB	T10	10	92	170	132.60	22.39
MTB		10	82	186	135.20	29.37

CTB	T11	10	106	178	146.00	22.35
MTB		10	90	194	146.80	29.34

CTB	T12	10	118	190	155.60	21.88
MTB		10	100	210	160.40	31.10

CTB	T13	10	130	202	166.20	21.96
MTB		10	106	220	171.40	32.84

CTB	T14	10	142	212	177.50	21.21
MTB		10	112	234	181.80	34.94

CTB	T15	10	158	224	188.20	20.41
MTB		10	118	248	192.80	36.08

CTB	T16	10	180	244	206.20	19.63
MTB		10	126	268	205.00	37.81

CTB	T17	10	192	250	223.20	19.19
MTB		10	130	288	215.80	40.63

CTB	T18	10	202	274	240.40	23.28
MTB		10	140	292	228.80	41.84

CTB	T19	10	212	288	257.20	25.23
MTB		10	155	302	240.50	41.99

CTB	T20	10	232	298	271.00	24.86
MTB		10	170	312	246.20	47.22

CTB	T21	10	250	312	286.20	26.08
MTB		10	112	320	244.80	63.77

CTB	T22	10	270	338	302.60	25.07
MTB		10	104	340	251.60	71.03

CTB	T23	10	280	352	315.80	27.91
MTB		10	98	368	252.60	82.00

CTB	T24	10	294	376	331.30	31.53
MTB		10	84	388	241.80	88.16

CTB	T25	10	274	390	334.50	43.43
MTB		10	73	400	241.40	96.61

CTB	T26	10	250	410	324.00	51.19
MTB		10	61	382	226.60	96.30

CTB	T27	10	231	389	300.00	52.26
MTB		10	42	366	207.20	97.51

CTB	T28	10	202	361	270.90	53.64
MTB		10	12	334	178.90	98.91

CTB	T29	10	0	348	88.50	144.56
MTB		10	0	308	52.00	111.94

CTB	T30	10	0	0	0.00	0.00
MTB		10	0	0	0.00	0.00

## Data Availability

The datasets used and/or analyzed during the current study are available from the corresponding author on reasonable request.
